# *Exophiala* Keratitis following Descemet Stripping Automated Endothelial Keratoplasty

**DOI:** 10.1155/2020/8872465

**Published:** 2020-10-29

**Authors:** Ana Marta, Paula Costa, Virgínia Lopes, Miguel Mesquita Neves, Miguel Gomes, Luís Oliveira

**Affiliations:** Department of Ophthalmology, Centro Hospitalar e Universitário do Porto, Oporto, Portugal

## Abstract

**Purpose:**

To report a case with *Exophiala spp.* keratitis in a Portuguese patient.

**Methods:**

A case report with deep corneal brown-pigmented infiltrates that developed 2 months after a Descemet Stripping Automated Endothelial Keratoplasty (DSAEK) for pseudophakic bullous keratopathy.

**Results:**

Diagnosis was established by positive direct examination and cultures from the surgically obtained corneal button. Slit-lamp images and anterior segment optical coherence tomography (AS-OCT) scans were obtained.

**Conclusion:**

This is the first described case of fungal keratitis caused by Exophiala spp. in Portugal and, to our knowledge, the first case following DSAEK in the literature.

## 1. Introduction

The black yeast genus *Exophiala* belongs to the order Chaetothyriales and represents over 40 different yeast species that occur as saprobes on plants [1]. *Exophiala xenobiotica* is a segregant of the *Exophiala jeanselmei* complex, frequently found in habitats rich in monoaromatic hydrocarbons and alkanes [2]. Eye infections due to *Exophiala s*pecies are extremely rare but have been reported [3].

Fungal keratitis is responsible for approximately 40% of all corneal ulcers in certain tropical climate countries but has a much lower incidence in temperate climates [1]. Fungal infection is an exceedingly rare complication following Descemet Stripping Automated Endothelial Keratoplasty (DSAEK), and it is always severe [4]. These infections tend to present in the late postoperative period as one or multiple infiltrates at the graft-host interface of the deep stroma. They are difficult to manage because the interface provides a unique sheltered environment [5].

We report a case that developed *Exophiala* spp. interface keratitis after DSAEK. This is the first described case of fungal keratitis caused by Exophiala spp. in Portugal and, to our knowledge, the first case following DSAEK in the literature.

## 2. Case Report

**Figure 1:**
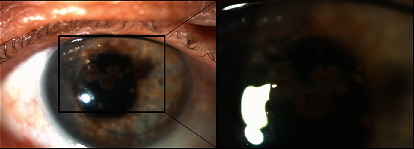
Slit-lamp image of the right eye at presentation. Dark multilobular lesions within deep stroma and a Descemet Stripping Automated Endothelial Keratoplasty graft as a subtle ring.

**Figure 2:**
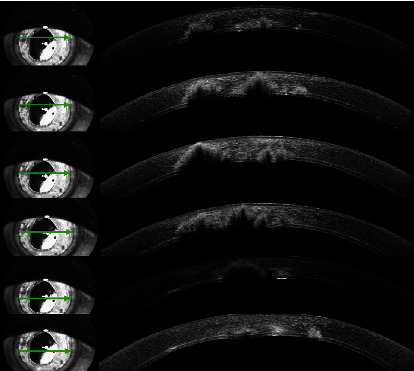
Anterior segment optical coherence tomography images of the patient at presentation. Graft-host interface and the stromal infiltrate with a shadow in the corneal stroma underlying the fungal lesion.

**Figure 3:**
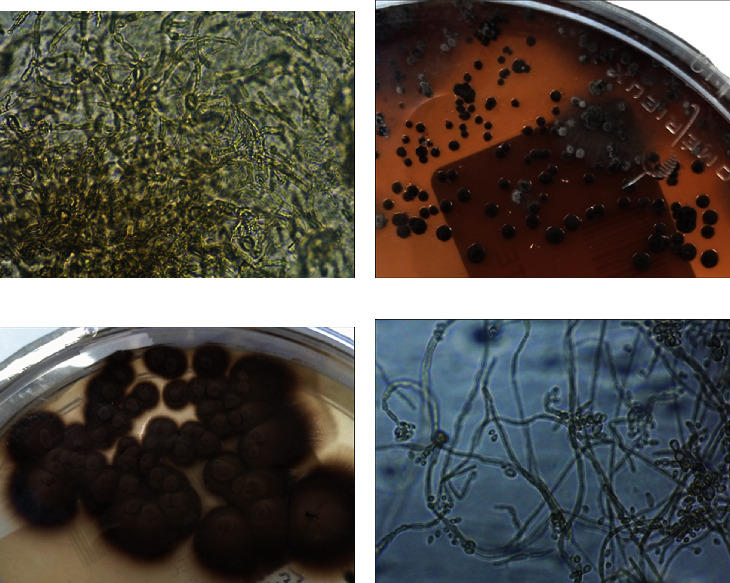
Microbiological examination. (a) Direct microscopy of ocular exudate: pigmented septate hyphae with some yeast cells. (b) Primary culture in blood agar medium and incubated at 37°C: black yeast colonies. (c) Primary culture in Sabouraud agar medium and incubated at 25°C: pigmented filamentous colonies. (d) Microscopic examination of the colonies.

A 68-year-old male, diagnosed with pseudophakic bullous keratopathy (PBK), underwent DSAEK in the right eye (RE). A postoperative follow-up was uneventful under a topical and systemic steroid regimen. The patient reported a history of eye infection caused by pine processionary (Thaumetopoea pityocampa), diagnosed and treated at a different hospital, 9 months before DSAEK. At the time of DSAEK, signs of eye infection were no longer reported. Two months after DSAEK, he was referred to our hospital due to keratitis on the RE (Figure 1). On examination, the best corrected visual acuity (BCVA) of the right eye (RE) was 20/200 and 20/20 in the LE. The pupils were isocoric and isoreactive, without afferent pupillary defect. He had no pain with ocular movements. The biomicroscopic examination of the RE revealed brown multilobular lesions within deep corneal stroma, with no conjunctival hyperaemia, secretions, or epithelial defect. The anterior chamber was deep and clear. Intraocular pressure and fundus examination were normal. The examination of his LE revealed no abnormality. An anterior segment optical coherence tomography (AS-OCT) (Spectralis®; Heidelberg Engineering, Heidelberg, Germany) was used to document the stromal infiltrate, showing the central infiltrate affecting all layers and the peripheral infiltrate concentrating in deep stroma (Figure 2). Given the spread and extent of the infection, an urgent surgical approach was chosen; therefore, no topical or systemic antifungal was performed before surgery. The patient underwent therapeutic penetrating keratoplasty (PK) using 8.0 mm diameter graft over a 7.75 mm patient's cornea trephination and 16 interrupted nylon 10/00 sutures, with no incident. After PK, the medical treatment used included standard approach, but with lower doses of systemic and topical corticosteroids due to suspected fungal etiology. The storage medium of the graft and patient's corneal button were sent to microbiologic examination. Diagnosis was established by a positive direct examination and culture (Figure 3) of the same fungus from the surgically obtained corneal button. There were no signs of recurrence of the infection during an extremely frequent initial follow-up; it was therefore decided not to medicate with antifungals, avoiding their associated toxicity. At 6 months of follow-up, no clinical signs of fungal infection developed and visual acuity was 20/200 without correction, but not all sutures had been removed.

## 3. Discussion

Few cases of keratitis caused by *Exophiala spp.* have been reported: *E. dermatitidis* keratitis after keratoplasty [6] and after laser in situ keratomileusis [7], *E. jeanselmei* keratitis after minor trauma [8, 9], and soft contact lens use [10] and *E. phaeomuriformis* keratitis after severe ocular surface problems [1]. *Exophiala xenobiotica* is a rare strain of *Exophiala*, and no case of keratitis caused by this agent has been previously reported. Common risk factors predisposing of mycotic keratitis are refractive contact lens wear, ocular surface disease, ocular trauma, and chronic topical corticosteroid therapy. Corneal pigmented lesions could also be caused by corneal melanoma or corneal melanin deposition caused by topical epinephrine therapy. The medical history can be important to form differential diagnoses.

In our case, the origin of the fungal infection remained unknown. Potential sources of this particular fungal organism in this patient are susceptibility to infectious keratitis due to steroid therapy after DASEK in a previously asymptomatic carrier: infected donor cornea or contamination of the graft from airborne particles, irrigation solutions, or instruments. The characteristics of AS-OCT suggest the lesions started in the posterior corneal layers, making infected donor cornea or contamination during graft manipulation the most likely causes. Despite the patient's history of Thaumetopoea pityocampa infection, this is an unlikely source, due to no association with *Exophiala xenobiotica* being reported and the absence of infection reported at the time of DSAEK. Microbiological analysis of the donor corneal rim could allow the diagnosis of donor cornea infection; however, this was not performed. In fact, routine culture of donor corneal rims remains controversial. Fungi, when isolated from donor corneal rims, have more risk to lead a clinical infection than bacteria [11]. Thus, the culture of the donor corneal rim (special fungal culture) in cases of lamellar or endothelial keratoplasty could allow for an early detection and treatment [12, 13].

Due to the generally poor response to medical treatment or of graft lenticule removal, we opted to perform a therapeutic penetrating keratoplasty.

This case highlights the possibility of corneal infection by less common organisms in temperate regions such as *Exophiala spp.* and in less likely situations. We should suspect a fungal infection if an infiltrate appears in the graft-host interface several weeks after DSAEK. Donor rim cultures are useful for more complete diagnosis as long as culture times should be long enough for fastidious organism to grow.

## Data Availability

Data supporting our study can be provided upon request.

## References

[1] Vicente A., Pedrosa Domellöf F., Byström B. (2018). *Exophiala phaeomuriformis* keratitis in a subarctic climate region: a case report. *Acta Ophthalmologica*.

[2] de Hoog G. S., Zeng J. S., Harrak M. J., Sutton D. A. (2006). *Exophiala xenobiotica* sp. nov., an opportunistic black yeast inhabiting environments rich in hydrocarbons. *Antonie Van Leeuwenhoek*.

[3] Homa M., Manikandan P., Saravanan V. (2018). *Exophiala dermatitidis* endophthalmitis: case report and literature review. *Mycopathologia*.

[4] Zhang Q., Randleman J. B., Stulting R. D. (2010). Clinicopathologic findings in failed descemet stripping automated endothelial keratoplasty. *Archives of Ophthalmology*.

[5] Palioura S., Sivaraman K., Joag M. (2018). Candida endophthalmitis after descemet stripping automated endothelial keratoplasty with grafts from both eyes of a donor with possible systemic candidiasis. *Cornea*.

[6] Benaoudia F., Assouline M., Pouliquen Y., Bouvet A., Guého E. (1999). *Exophiala (Wangiella) dermatitidis* keratitis after keratoplasty. *Medical Mycology*.

[7] Patel S. R., Hammersmith K. M., Rapuano C. J., Cohen E. J. (2006). Exophiala dermatitidis keratitis after laser in situ keratomileusis. *Journal of Cataract and Refractive Surgery*.

[8] Saeedi O. J., Iyer S. A., Mohiuddin A. Z., Hogan R. N. (2013). Exophiala jeanselmei keratitis. *Eye & Contact Lens*.

[9] Ben-Simon G. J., Grinbaum A., Barequet I. S. (2002). More than tears in your eyes (Exophiala jeanselmei keratitis). *Cornea*.

[10] Hay J., Patel S., Seal D. V. (1995). Exophiala Jeanselmei: a potential ocular pathogen. *Journal of the British Contact Lens Association.*.

[11] McElnea E., Power B., Murphy C. (2018). Interface fungal keratitis after descemet stripping automated endothelial keratoplasty: a review of the literature with a focus on outcomes. *Cornea*.

[12] Augustin V. A., Weller J. M., Kruse F. E., Tourtas T. (2018). Fungal interface keratitis after descemet membrane endothelial keratoplasty. *Cornea*.

[13] Nahum Y., Russo C., Madi S., Busin M. (2014). Interface infection after descemet stripping automated endothelial keratoplasty: outcomes of therapeutic keratoplasty. *Cornea*.

